# Should online, free proctology videos be used for self‐directed post‐graduate learning? A proposed evaluation using a colorectal video assessment framework

**DOI:** 10.1111/codi.70313

**Published:** 2025-11-16

**Authors:** Shoeib Mirdha, Evripidis Tokidis, Louise Le Blevec, Tim Wilson

**Affiliations:** ^1^ Doncaster and Bassetlaw Teaching Hospitals NHS Foundation Trust Doncaster UK; ^2^ Department of Clinical Medicine, School of Medicine and Population Health The University of Sheffield Sheffield UK

**Keywords:** proctology, recorded procedural videos, self‐directed learning, YouTube™

## Abstract

**Introduction:**

Exposure to proctology during post‐graduate colorectal training is often variable. Videos of proctological procedures can benefit surgical trainees' self‐directed learning. The aim of this study is to evaluate the quality of freely available online video material on proctological procedures using a modified colorectal video assessment framework.

**Methods:**

PubMed and the YouTube™ platform were searched for the following terms related to proctological procedures for haemorrhoids, anal fissure and fistula. These were assessed (cross‐sectional study) for quality using a modified video‐assessment checklist that was validated by three colorectal surgeons who regularly perform proctology cases. The resulting 9‐item evaluation tool was designed to capture the extent to which videos provide concise and structured information typically required for peer review.

**Results:**

A total of 98 surgical videos were assessed, comprising 65 from peer‐reviewed. Journals and 35 from YouTube™ only. The median total score for peer‐reviewed videos was 16.0 (interquartile range [IQR] 13.0–17.0) compared to 10 (IQR 8.0–12.0) for the non‐peer‐reviewed videos. This difference was statistically significant (Mann–Whitney *U* = 2024.0, *p* < 0.001). In particular, journal videos were significantly better at providing more contextual information about the case including presenting symptoms and outcomes.

**Conclusion:**

As might be expected, the quality of YouTube™ videos from the perspective of proctology training was inferior to those released online by peer‐reviewed journals. This provides further evidence for the validity of using modified checklists to assess the quality of training materials. Given the findings of this study, trainees should be encouraged to prioritise journal‐related over other freely available material for self‐directed learning.

**What does this paper add to the literature?**

This study demonstrates that a modified, face‐validated checklist can assess the educational quality of proctology videos, showing that peer‐reviewed journal content significantly outperforms non‐peer‐reviewed, freely available YouTube videos, and providing empirical evidence to guide trainees towards peer‐reviewed resources.

## INTRODUCTION

Proctology constitutes a recognised subspecialty within colorectal surgery and encompasses the diagnosis and operative management of benign anorectal conditions, including haemorrhoids, anal fissures and anal fistulae [1]. Despite the frequency and clinical significance of these conditions, structured training in proctology during colorectal postgraduate education is often inconsistently implemented across training programmes. In particular, early exposure to proctological procedures is often absent, with many trainees encountering such cases only in the latter stages of their specialty training or during dedicated fellowships [2, 3].

In contemporary surgical education, there is an expanding pool of supplementary resources to support ‘self‐directed learning’ [4], particularly freely available online surgical videos [5, 6]. These videos serve as preparatory material prior to assisting or performing operative procedures and may be particularly valuable in low‐volume or subspecialty areas such as proctology. However, the educational value of such video‐based learning is contingent upon the unregulated quality and fidelity of the content [7].

Recognising this, efforts have been made to create standardised frameworks to assess and improve the quality of educational surgical videos [8]. One such framework is the Laparoscopic Video Educational Guidelines and Score (LAP‐VEGaS), developed through expert consensus among colorectal surgeons to evaluate the quality of educational laparoscopic colorectal surgical videos submitted for peer review [9]. To date, however, no such validated assessment framework exists specifically for proctological procedures, despite the widespread availability of related content online.

The aim of this study was to use a modified LAP‐VEGaS checklist to compare the quality of proctological training videos hosted on the YouTube™ platform to video vignettes published by peer‐reviewed journals.

## METHODS

**FIGURE 1:**
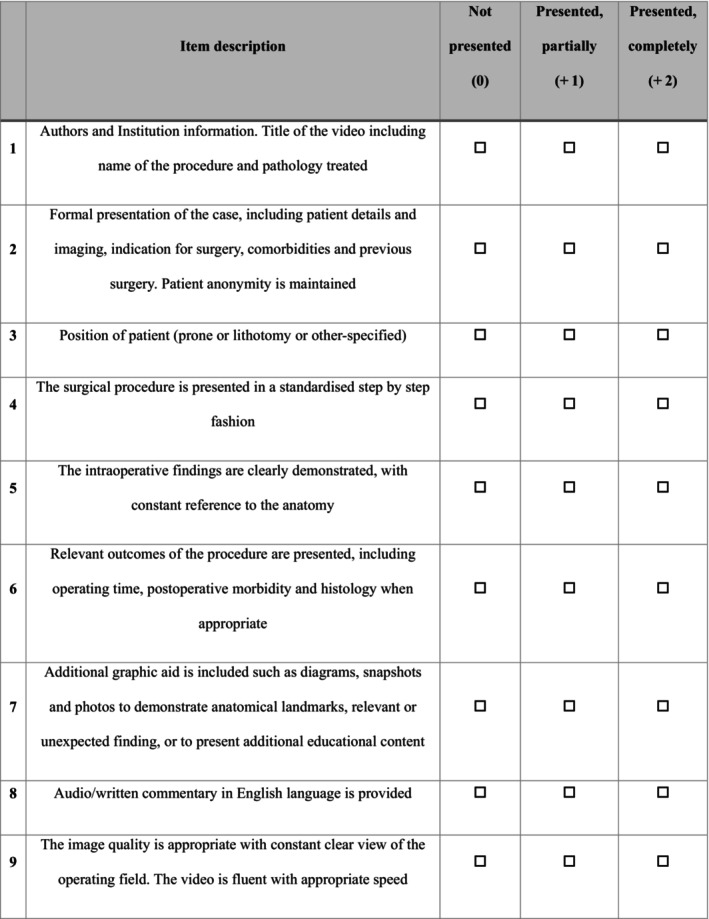
Modified proctology video‐assessment tool.

The LAP‐VEGaS video assessment tool [9] was reviewed and adapted for the specific context of proctological procedures. Three consultant colorectal surgeons with regular proctology practice were asked to review each criterion of the LAP‐VEGaS checklist to determine its relevance and applicability to open and minimally invasive proctological operations. Based on feedback, modifications were made to enhance the checklist's suitability for assessing the educational quality of proctology‐specific video content. For the purposes of this study, all items were equally weighted to ensure simplicity and reproducibility. Items specific to laparoscopic access and port placement were removed, while items such as patient positioning, case presentation and outcome reporting were retained and adapted for proctology, resulting in a 9‐item checklist (Figure 1).

Figure 1 shows the final face‐validated framework, which was used as the primary instrument for subsequent video evaluation. Each of its nine items was scored on a 3‐point ordinal scale (0 = not presented, 1 = partially presented, 2 = completely presented), yielding a maximum possible total score of 18 (calculated as the sum of the score of all nine items). As a reference point, a total score closer to 18 indicates greater adherence to the checklist criteria and may be interpreted as more complete educational content. While the original LAP‐VEGaS validation did not define absolute thresholds for poor, moderate or good quality, higher cumulative scores have been used as proxies for educational adequacy in prior studies evaluating laparoscopic colorectal videos.

On 18 March 2025, the YouTube™ platform was searched for videos on proctology procedures. The search was revisited on 31 May 2025 to ensure reliability of the findings. Both searches were conducted using an institutional computer with the browser set to private (incognito) mode, to minimise the potential influence of personalised search algorithms or prior user activity. Following a systematic approach, the videos were filtered based on view count, and the 85 most viewed were subsequently subjected to independent analysis [10].

The following terms were searched on YouTube™ platform (no date restrictions have been applied): haemorrhoids, fissure and fistula. These capture the commonest perianal conditions that general surgery trainees with a colorectal interest are expected to develop competency in [11]. Similarly, in PubMed, we searched the following string: (video OR vign*) AND proctology AND (haemorrhoid* OR fissure OR fistula*). These were screened between two reviewers (ET and SM) to match the inclusion criteria.

**FIGURE 2:**
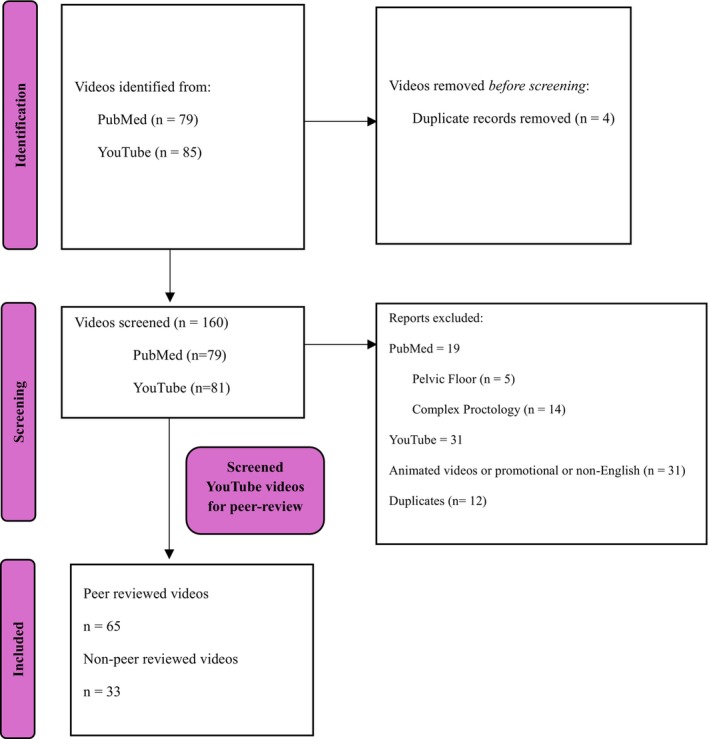
Video inclusion flow diagram adapted from Page MJ, et al. BMJ 2021;372:n71. https://doi.org/10.1136/bmj.n71.

Videos were excluded if duplicate, non‐English, animated, promotional or addressing complex proctology/pelvic floor procedures, to better reflect the material relevant to a typical proctology training list. The videos were then categorised as ‘peer‐reviewed’ and ‘non‐peer‐reviewed’ as journals also publish their peer‐reviewed video material on YouTube™ (Figure 2). The included videos on proctology procedures were assessed by two researchers (ET, SM) against the revised tool (Tables S1 and S2). Videos were assessed independently by the two authors; no screening conflicts were identified, and complete agreement was observed across all ratings.

The data were recorded on a pre‐defined Microsoft Excel [12] proforma, and further analysis was performed using the Jamovi open access platform [13]. This was an observational cross‐sectional study that adhered to the STROBE (Strengthening the Reporting of Observational Studies in Epidemiology) guidelines (STROBE checklist). A comparative analysis was conducted between 33 non‐peer‐reviewed and 65 peer‐reviewed proctology videos. Descriptive statistics summarised total and item‐specific scores. As the data were not normally distributed, the non‐parametric Mann–Whitney *U*‐test was used to compare total scores between the YouTube™ and PubMed groups.

## RESULTS

A total of 98 surgical videos demonstrating various proctological procedures were included in the final analysis. Of these, 65 videos (66.3%) were peer‐reviewed videos and 33 (33.7%) were not peer‐reviewed. Each video was evaluated using a 9‐item structured assessment tool adapted for proctology as described in the methods. Peer‐reviewed videos demonstrated overall superiority to those that were not peer‐reviewed. The median total score for peer‐reviewed videos was 16.0 [interquartile range (IQR) 13.0–17.0] compared to 10 (IQR 8.0–12.0) for the non‐peer‐reviewed videos. This difference was statistically significant (Mann–Whitney *U* = 2024.0, *p* < 0.001). The total scores were also more consistent for peer‐reviewed videos, with a range of 7–18, compared to 0–16 for the non‐peer‐reviewed group.

Across the nine individual assessment items, non‐peer‐reviewed videos were more frequently assigned lower scores. Lower item scores in this group were most observed in those components assessing the formal presentation of the clinical case, reporting of procedural outcomes, specification of intraoperative positioning and the inclusion of visual or graphic aids. Scores for items relating to the structured depiction of operative steps, reference to relevant anatomical landmarks, the presence of English‐language audio or written commentary, and image quality were generally higher, though variation remained between platforms. Although some items had equal median scores across groups, the Mann–Whitney *U*‐test identified significant differences due to variation in the overall distribution of scores. Overall, non‐peer‐reviewed videos scored lower across most items, particularly for case presentation, outcomes, intraoperative positioning and use of visual aids, whereas both sources performed similarly for technical depiction, anatomy, commentary and image quality (Table 1).

**TABLE 1:** Median scores for each checklist item (0–2 scale; higher values indicate more complete educational content); *p*‐values from Mann–Whitney *U*‐test comparing peer‐reviewed to non‐peer‐reviewed videos.

Item	Median (peer‐reviewed videos)	Median (non‐peer‐reviewed videos)	*p*‐value
1. Authors and Institution information. Title of the video including name of the procedure and pathology treated	2.0	1.0	0.002
2. Formal presentation of the case, including patient details and imaging, indication for surgery, comorbidities and previous surgery. Patient anonymity is maintained	2.0	1.0	<0.001
3. Position of patient (prone or lithotomy or other‐specified)	2.0	1.0	<0.001
4. The surgical procedure is presented in a standardised step by step fashion	2.0	2.0	0.001
5. The intraoperative findings are clearly demonstrated, with constant reference to the anatomy	2.0	2.0	0.007
6. Relevant outcomes of the procedure are presented, including operating time, postoperative morbidity and histology when appropriate	1.0	0.0	0.001
7. Additional graphic aid is included such as diagrams, snapshots and photos to demonstrate anatomical landmarks, relevant or unexpected finding, or to present additional educational content	1.0	1.0	0.294
8. Audio/written commentary in English language is provided	2.0	2.0	0.453
9. The image quality is appropriate with constant clear view of the operating field. The video is fluent with appropriate speed	2.0	2.0	0.244
Total Item Score	16	10	<0.001

## DISCUSSION

This study applied a modified video assessment framework, derived from the LAP‐VEGaS tool to peer and non‐peer‐reviewed videos. Consistent omissions were observed across the data set. The majority of non‐peer‐reviewed YouTube™ videos lacked information on preoperative work‐up, intra‐operative set‐up, equipment used and postoperative outcomes. These elements are critical in the context of surgery and the absence of preoperative work‐up may obscure crucial considerations, such as patient comorbidities, imaging findings or indications for surgery [14]. Similarly, the omission of intraoperative set‐up and equipment specification undermines the reproducibility and educational value of the video, given the procedural nuances and specialised instrumentation often required in proctology [1]. The absence of postoperative outcomes omits key information that might limit the understanding of the procedures' impact.

Educational elements such as diagrams, tables and commentary on clinical context were infrequently included. This is important in proctology, where operative fields are often confined, anatomical planes are subtle, and variations in pathology presentation may influence technical decision‐making [15, 16]. Visual aids and structured narration serve to enhance cognitive assimilation, especially for learners unfamiliar with the nuanced anatomy of the anorectal region [17]. Furthermore, commentary that situates the procedure within a broader clinical framework—such as indications, contraindications and differential diagnoses—may augment the didactic value of the video by facilitating integration with theoretical knowledge and evidence‐based practice [18].

Conversely, procedural steps and anatomical demonstration were present in most videos regardless of platform. This is a notable strength, as the clear display of operative technique and anatomical structures is foundational to surgical education. Accurate anatomical demonstration supports spatial understanding, while stepwise procedural depiction aids procedural memory and cognitive rehearsal [8]. In the context of proctology, where tactile feedback and limited visual exposure during real‐life operations pose significant training challenges, video‐based reinforcement of technique and anatomy provides an accessible and potentially scalable supplementary tool for trainees.

In summary, the findings of this study are consistent with previous evaluations of online surgical videos, which have reported limited case detail, poor procedural context and minimal outcome reporting, particularly in non‐peer‐reviewed content [7]. These observations align with Sturiale et al., who demonstrated that a substantial proportion of YouTube™ videos on haemorrhoid disease were inaccurate or educationally inadequate, reinforcing concerns regarding the reliability of freely available surgical content [19]. While journal‐hosted videos demonstrated superior quality, it should be recognised that YouTube™ may still serve to widen access for trainees without full journal subscriptions.

No prior tool has been identified for the specific assessment of proctology‐related surgical videos. The modified framework used here is based on an existing validated tool developed for laparoscopic colorectal surgery and was adapted through consensus among colorectal surgeons with regular experience in proctology and the findings of this study provide some degree of additional validation for the usefulness of this tool. Further collaborative research could evaluate the reproducibility of the modified tool and explore its utility in guiding the development of proctology‐specific educationally relevant video resources. Content creators of such material may also be encouraged to adopt this framework.

## LIMITATIONS

The initial face validity of the assessment tool was performed within a single institution in South Yorkshire, United Kingdom, which may restrict the generalisability of the findings. Furthermore, the analysis was confined to videos available on YouTube™ and PubMed, potentially introducing selection bias and limiting the representativeness of the sample. The selection of YouTube™ videos based on view count, whereas consistent with prior studies [10], may introduce bias, as popularity does not necessarily equate to educational quality. A small number of videos outside the three predefined search terms were retained in the YouTube™ sample to preserve the methodology of selecting the 50 most viewed videos; this may have introduced minor heterogeneity. Subgroup analyses by factors such as country of origin or date of publication were not performed, which may limit further contextual interpretation. Additionally, search results are influenced by algorithmic factors and are temporally dynamic. While independent assessment by two reviewers aimed to enhance objectivity, the resolution of discrepancies through consensus may still be subject to interpretive bias, and the study did not evaluate the educational relevance or accuracy of the video content.

## CONCLUSION

This study demonstrates that freely available YouTube™ videos of proctological procedures may be less useful training tools than peer‐reviewed videos identified through PubMed. This should be kept in mind while using freely available videos for learning purposes and improving clinical practice along with surgical skills.

## AUTHOR CONTRIBUTIONS

**Shoeib Mirdha:** Data curation; investigation; validation; formal analysis; writing – original draft; visualization; conceptualization; methodology; writing – review and editing; project administration. **Evripidis Tokidis:** Conceptualization; methodology; software; data curation; investigation; validation; supervision; project administration; writing – review and editing; visualization; resources. **Louise Le Blevec:** Investigation; validation; data curation; project administration. **Tim Wilson:** Supervision; writing – review and editing.

## FUNDING INFORMATION

The authors declare that they did not receive any funding for this study.

## CONFLICT OF INTEREST STATEMENT

The authors declare no conflict of interest.

## ETHICS STATEMENT

This study, involving the analysis of publicly available online videos, was conducted in accordance with the principles of the Declaration of Helsinki; formal ethics approval was not required.

## Supporting information


Table S1.
Table S2.

## Data Availability

The data that support the findings of this study are available from the corresponding author upon reasonable request.
