# Supplementary material for: Should online, free proctology videos be used for self‐directed post‐graduate learning? A proposed evaluation using a colorectal video assessment framework
Source: Colorectal Dis. 2025 Nov 16;27(11):e70313. doi: 10.1111/codi.70313 (PMC12620539; doi:10.1111/codi.70313)
Supplement: Supplementary file 1 — Table S1. Table S2. [file CODI-27-0-s001.docx]

| **Video Links** | **Video names** | **Topic** | **Item 1** | **Item 2** | **Item 3** | **Item 4** | **Item 5** | **Item 6** | **Item 7** | **Item 8** | **Item 9** |
| --- | --- | --- | --- | --- | --- | --- | --- | --- | --- | --- | --- |
| 10.1111/codi.17216 | Postoperative analgesia post-haemorrhoidectomy with bilateral pudendal block guided by neurostimulator-a video vignette | Local anaesthesia technique | 2 | 2 | 2 | 2 | 2 | 2 | 2 | 2 | 2 |
| 10.1111/codi.17190 | Digital platform for the treatment of II-III degree haemorrhoidal disease with 3% polidocanol foam: A video vignette | Haemorrhoids | 2 | 1 | 2 | 2 | 2 | 2 | 2 | 2 | 2 |
| 10.1111/codi.17060 | SHAPE-Skin-sparing haemorrhoidectomy and pexy: A video vignette | Haemorrhoids | 2 | 2 | 2 | 2 | 2 | 2 | 2 | 2 | 2 |
| 10.1111/codi.17009 | Seamless open excisional haemorrhoidectomy-Six faces of surgical technique: A Video Vignette | Haemorrhoids | 2 | 2 | 2 | 2 | 2 | 2 | 2 | 2 | 2 |
| 10.1111/codi.16892 | Implementation of video-assisted anal fistula treatment (VAAFT) for complex fistulas - A Video Vignette | Fistula | 2 | 2 | 2 | 2 | 2 | 2 | 2 | 2 | 2 |
| 10.1111/codi.16792 | Side-to-side anal flap for the surgical treatment of low trans-sphincteric fistula-in-ano-A video vignette | Fistula | 2 | 2 | 2 | 2 | 2 | 2 | 2 | 2 | 2 |
| 10.1111/codi.16758 | Complex perianal fistula in a patient with Crohn's disease treated with fistula laser closure therapy - A video vignette | Fistula | 2 | 2 | 2 | 2 | 2 | 2 | 0 | 2 | 2 |
| 10.1111/codi.16557 | Ligation of the intersphincteric fistula tract: a video vignette | Fistula | 2 | 2 | 2 | 2 | 2 | 2 | 0 | 2 | 2 |
| 10.1111/codi.16504 | Autological platelet-rich fibrin sealant for anterior horseshoe anal fistula in female patient: A video vignette | Fistula | 2 | 2 | 2 | 2 | 2 | 2 | 0 | 2 | 2 |
| 10.1111/codi.16405 | Radiofrequency ablation for haemorrhoids-A video vignette | Haemorrhoids | 2 | 2 | 2 | 2 | 2 | 2 | 0 | 2 | 2 |
| 10.1111/codi.16338 | Tailored mucosectomy and haemorrhoidopexy (TM & H): A new surgical option for selected Goligher grade III haemorrhoids - a video vignette | Haemorrhoids | 2 | 2 | 2 | 2 | 2 | 2 | 2 | 2 | 2 |
| 10.1111/codi.16311 | Adult allogeneic bone-marrow-derived mesenchymal stem cells for the treatment of anovaginal and perianal fistulas in the setting of Crohn's like phenotype of the ileal pouch-anal anastomosis - a video vignette | Fistula | 2 | 2 | 2 | 2 | 2 | 1 | 2 | 2 | 2 |
| 10.1111/codi.16250 | Treatment of horseshoe fistula with a two-stage modified Hanley procedure: A video vignette | Fistula | 2 | 2 | 2 | 2 | 2 | 0 | 2 | 2 | 2 |
| 10.1111/codi.16206 | Mesenchymal stem cells for the treatment of perianal fistulizing Crohn's disease-A video vignette | Fistula | 2 | 1 | 2 | 2 | 1 | 1 | 1 | 1 | 1 |
| 10.1111/codi.16137 | Injection of mesenchymal stem cells (darvadstrocel) into Crohn's perianal fistula - A video vignette | Fistula | 2 | 2 | 2 | 2 | 2 | 2 | 1 | 2 | 2 |
| 10.1111/codi.16134 | THD Anolift for advanced haemorrhoidal disease: a video vignette | Haemorrhoids | 1 | 1 | 2 | 2 | 2 | 2 | 2 | 2 | 2 |
| 10.1111/codi.16044 | Marsupialization of fistulotomy wound in an intersphincteric fistula - A Video Vignette | Fistula | 2 | 2 | 2 | 2 | 2 | 2 | 2 | 2 | 2 |
| 10.1111/codi.15992 | The use of a new automated device for the sclerosing treatment of haemorrhoidal disease - A video-vignette | Haemorrhoids | 2 | 0 | 1 | 2 | 2 | 2 | 2 | 2 | 2 |
| 10.1111/codi.15900 | Lateral internal sphincterotomy (LIS) using endoanal ultrasound - a video vignette | Fissure | 2 | 2 | 2 | 2 | 2 | 2 | 1 | 2 | 2 |
| 10.1111/codi.15749 | Easy, safe and fast knot tying technical variation ('siphoning') in the transanal haemorrhoidal dearterialization procedure - a video vignette | Haemorrhoids | 2 | 2 | 2 | 2 | 2 | 2 | 2 | 2 | 2 |
| 10.1111/codi.15745 | Fistulotomy plus end-to-end primary sphincteroplasty - a video vignette | Fistula | 2 | 2 | 2 | 2 | 2 | 2 | 0 | 2 | 2 |
| 10.1111/codi.15742 | Stem cell therapy for anal fistula treatment - a video vignette | Fistula | 2 | 1 | 2 | 1 | 2 | 2 | 0 | 2 | 2 |
| 10.1111/codi.15627 | Fistula closure by laser - a video vignette | Fistula | 2 | 2 | 2 | 2 | 2 | 2 | 2 | 2 | 2 |
| 10.1111/codi.15613 | Combined rubber band ligation with 3% polidocanol foam sclerotherapy (ScleroBanding) for the treatment of second-degree haemorrhoidal disease: a video vignette | Haemorrhoids | 2 | 2 | 2 | 2 | 2 | 2 | 1 | 2 | 2 |
| 10.1111/codi.15563 | Rectal advancement flap for complex anal fistula-a video vignette | Fistula | 2 | 2 | 2 | 2 | 2 | 1 | 2 | 2 | 2 |
| 10.1111/codi.15540 | Radiofrequency haemorrhoidectomy: technical guide to our standardized technique - a video vignette | Haemorrhoids | 2 | 1 | 2 | 2 | 2 | 0 | 1 | 2 | 2 |
| 10.1111/codi.15264 | Anal fistula laser ablation - a video vignette | Fistula | 2 | 2 | 2 | 2 | 2 | 1 | 2 | 2 | 2 |
| 10.1111/codi.15226 | Single-stapled parachute technique for grade IV prolapsed haemorrhoids - a video vignette | Haemorrhoids | 1 | 2 | 2 | 2 | 2 | 1 | 1 | 2 | 2 |
| 10.1111/codi.15194 | Treatment of recurrent anterior transsphincteric fistula with fistula plug - a video vignette | Fistula | 1 | 2 | 2 | 2 | 2 | 0 | 0 | 2 | 2 |
| 10.1111/codi.15178 | A tailored rhomboid advancement flap for severe anal stenosis - a video vignette | Anal stenosis | 2 | 0 | 2 | 2 | 2 | 0 | 0 | 2 | 2 |
| 10.1111/codi.15096 | The LIFT (ligation of the intersphincteric fistula tract) procedure for a transsphincteric posterior anal fistula - a video vignette | Fistula | 1 | 2 | 2 | 2 | 2 | 0 | 2 | 2 | 2 |
| 10.1111/codi.15061 | Step-by-step open excisional haemorrhoidectomy for grade IV circular haemorrhoidal disease - a video vignette | Haemorrhoids | 2 | 2 | 2 | 2 | 2 | 1 | 1 | 2 | 2 |
| 10.1111/codi.15067 | Fistulotomy for intersphincteric fistula. A stepwise approach for surgical trainees - a video vignette | Fistula | 1 | 1 | 2 | 2 | 2 | 0 | 2 | 2 | 2 |
| 10.1111/codi.15071 | Intra-anal fistulotomy with marsupialization for recurrent high intersphincteric fistula - a video vignette | Haemorrhoids | 1 | 2 | 2 | 2 | 2 | 2 | 2 | 2 | 2 |
| 10.1111/codi.15050 | Video-assisted anal fistula treatment in combination with ligation of the intersphincteric fistula tract in the treatment of complex transsphincteric fistulas - a video vignette | Fistula | 2 | 2 | 2 | 2 | 2 | 2 | 0 | 2 | 2 |
| 10.1111/codi.15040 | Use of video-guided sclerotherapy with 3% polidocanol foam for symptomatic second-degree haemorrhoidal disease - a video vignette | Haemorrhoids | 2 | 1 | 2 | 2 | 1 | 0 | 1 | 2 | 2 |
| 10.1111/codi.14937 | Rectal full-thickness advancement flap for the treatment of high transsphincteric fistula-in-ano - a video vignette | Fistula | 1 | 1 | 2 | 1 | 1 | 0 | 0 | 2 | 2 |
| 10.1111/codi.14941 | Lateral internal sphincterotomy for chronic anal fissure under perianal anaesthetic infiltration - a video vignette | Fissure | 1 | 2 | 2 | 2 | 1 | 0 | 0 | 2 | 1 |
| 10.1111/codi.14934 | External anal sphincter sparing seton after rerouting of the fistula tract - a video vignette | Fistula | 2 | 0 | 2 | 2 | 2 | 0 | 0 | 2 | 2 |
| 10.1111/codi.14935 | LigaSure haemorrhoidectomy - a technical guide - a video vignette | Haemorrhoids | 1 | 1 | 2 | 2 | 1 | 0 | 0 | 2 | 2 |
| 10.1111/codi.14839 | Permacol™ collagen paste injection for the treatment of complex anal fistula - a video vignette | Fistula | 1 | 1 | 1 | 1 | 1 | 0 | 0 | 1 | 1 |
| 10.1111/codi.14827 | A step-by-step demonstration of Whitehead's haemorrhoidectomy for trainees - a video vignette | Haemorrhoids | 2 | 2 | 1 | 2 | 2 | 0 | 0 | 2 | 2 |
| 10.1111/codi.14823 | Video-assisted anal fistula treatment (VAAFT) assisted seton placement - a video vignette | Fistula | 2 | 1 | 1 | 2 | 1 | 0 | 1 | 2 | 2 |
| 10.1111/codi.14660 | The LIFT procedure for a perianal Crohn's fistula - a video vignette | Fistula | 1 | 1 | 2 | 1 | 1 | 0 | 0 | 2 | 2 |
| 10.1111/codi.14663 | Injection of freshly collected autologous adipose tissue for treatment of perianal fistula in a patient with Crohn's disease - a video vignette | Fistula | 2 | 2 | 2 | 2 | 2 | 1 | 1 | 2 | 2 |
| 10.1111/codi.14656 | Scissor haemorrhoidectomy under perineal anaesthetic infiltration as a day case procedure - a video vignette | Haemorrhoids | 0 | 1 | 2 | 2 | 1 | 0 | 0 | 2 | 1 |
| 10.1111/codi.14551 | The use of 3D imaging to facilitate training during complex fistula surgery - a video vignette | Fistula | 2 | 2 | 2 | 2 | 2 | 1 | 2 | 2 | 2 |
| 10.1111/codi.14531 | Autotransplantation of autologous adipose tissue-derived mesenchymal stem cells to treat complex fistula-in-ano - the FLiRT technique - a video vignette | Fistula | 1 | 1 | 2 | 2 | 2 | 1 | 0 | 2 | 2 |
| 10.1111/codi.14498 | The use of sclerotherapy with polidocanol foam in the treatment of second-degree haemorrhoidal disease - a video vignette | Haemorrhoids | 2 | 1 | 1 | 1 | 1 | 0 | 1 | 2 | 2 |
| 10.1111/codi.14496 | First aid toolkit for anal fistulas, a detailed treatise for trainees - a video vignette (ESCP Trainee Video) | Fistula | 2 | 2 | 1 | 2 | 2 | 1 | 2 | 2 | 2 |
| 10.1111/codi.14495 | Treatment of perianal sepsis, a detailed treatise for trainees - a video vignette (ESCP Trainee Video) | Sepsis | 2 | 2 | 2 | 2 | 2 | 1 | 2 | 2 | 2 |
| 10.1111/codi.14459 | Transanal advancement flap repair; step-by-step guide for trainees - a video vignette (ESCP trainee video) | Fistula | 2 | 2 | 2 | 2 | 2 | 2 | 1 | 2 | 2 |
| 10.1111/codi.14429 | Ligation of intersphincteric fistula tract procedure for the treatment of fistula in ano - a video vignette | Fistula | 2 | 2 | 2 | 2 | 2 | 1 | 2 | 2 | 2 |
| 10.1111/codi.14261 | A simplified easily reproducible pudendal nerve block technique for anorectal surgery (SEPTA) - a video vignette | Local anaesthesia technique | 0 | 1 | 1 | 2 | 1 | 0 | 1 | 2 | 2 |
| 10.1111/codi.14217 | Surgical anatomy of the deep postanal space and the re-modified Hanley procedure - a video vignette | Anatomy | 2 | 2 | 2 | 2 | 2 | 1 | 2 | 2 | 2 |
| 10.1111/codi.13415 | Surgical management of complex fistulizing Crohn's disease - a video vignette | Fistula | 2 | 2 | 2 | 2 | 2 | 2 | 2 | 2 | 2 |
| 10.1111/codi.13143 | Tube in tract technique: a simple alternative to a loose draining seton in the management of complex fistula-in-ano--a video vignette | Fistula | 0 | 0 | 2 | 2 | 2 | 0 | 1 | 2 | 2 |
| 10.1111/codi.12918 | The vertical rectus abdominis myocutaneous flap--a video vignette | Fistula | 2 | 2 | 1 | 2 | 2 | 0 | 0 | 2 | 2 |
| 10.1111/codi.12799 | Robotic transanal fistula repair - a video vignette | Fistula | 2 | 2 | 2 | 2 | 2 | 0 | 2 | 2 | 1 |
| <https://youtu.be/X-Qh6iAK4Ic?si=0EVHMuGG_k3Qpg4t> (DCR) | Excisional haemorrhoidectomy: closed technique | Haemorrhoids | 2 | 2 | 2 | 2 | 2 | 0 | 2 | 2 | 2 |
| <https://youtu.be/RU2PHNPfVXk?si=cWJdf5ICDjcax4-x> (CODI) | Laser hemorrhoidoplasty | Haemorrhoids | 0 | 1 | 1 | 2 | 2 | 1 | 0 | 2 | 2 |
| <https://youtu.be/sZgJokkU5dg?si=p_FfL0oLmmn3Bm4W> (CODI) | Laser hemorrhoidoplasty procedure (LHP) for grade 2 haemorrhoidal disease | Haemorrhoids | 2 | 2 | 1 | 2 | 2 | 1 | 0 | 2 | 2 |
| <https://youtu.be/HEdR7lCq7NA?si=vIUaRwgnaJudL927> (CODI) | A Technical note to the treatment of large perianal condylomata acuminata | Wart | 2 | 2 | 2 | 2 | 2 | 1 | 2 | 2 | 2 |
| <https://youtu.be/6nKUuNQ2kuc?si=ZWoi4gKcIly4wQ8S> (CODI) | Fistula Laser ablation therapy (Filac@)—a video vignette | Fistula | 2 | 2 | 2 | 2 | 2 | 1 | 1 | 2 | 2 |
| <https://www.youtube.com/watch?v=g28akcpcWoQ> (CODI) | Transanal Suture Mucopexy - a non-excisional cost-effective procedure for advanced hemorrhoids | Haemorrhoids | 1 | 2 | 2 | 2 | 1 | 0 | 0 | 2 | 2 |
| <https://youtu.be/hX2nG3LoOYs?si=E5uozq7wUvi6bMX2> | Surgical management of giant anorectal condyloma by local excision and V-Y anoplasty | Wart | 2 | 2 | 2 | 2 | 2 | 1 | 2 | 2 | 2 |
| <https://youtu.be/ZZXcAadMGRc?si=_LQoHLo7Yu6MXgW-> | Transanal excision of giant rectal polyp | Polyp | 2 | 1 | 1 | 1 | 1 | 1 | 0 | 2 | 2 |
| <https://youtu.be/di0y4wdx064?si=ToKqvWeOj7T1p2Tl> | Hybrid transanal resection of near-circumferential large, low rectal polyp | Polyp | 2 | 2 | 2 | 2 | 2 | 2 | 2 | 2 | 2 |
| <https://youtu.be/dqVauLWgZ5k?si=HK7FqeBuvaOaJ27H> | Rectal advancement flap for the treatment of fistula-in-ano | Fistula | 2 | 2 | 1 | 2 | 2 | 0 | 1 | 2 | 2 |

**Table S1: Peer reviewed videos along with scoring using modified assessment tools**

| **Video Links** | **Name of the videos** | **Topic** | **Item 1** | **Item 2** | **Item 3** | **Item 4** | **Item 5** | **Item 6** | **Item 7** | **Item 8** | **Item 9** |
| --- | --- | --- | --- | --- | --- | --- | --- | --- | --- | --- | --- |
| <https://youtu.be/m01dM1BLYgw?si=z2DGZQFBxt83MdUw> | Diathermy excisional haemorrhoidectomy: still the gold standard | Haemorrhoids | 1 | 1 | 1 | 2 | 2 | 0 | 1 | 2 | 2 |
| <https://youtu.be/1Dv_xk8zC5w?si=n_Tr9uv2mEkePBRh> | Haemorrhoidectomy | Haemorrhoids | 2 | 1 | 1 | 2 | 2 | 0 | 1 | 2 | 2 |
| <https://youtu.be/wdliRML1jm4?si=pcQn0rkv8MEFDZTN> | Milligan Morgan haemorrhoidectomy for 3rd degree haemorrhoids | Haemorrhoids | 1 | 1 | 2 | 2 | 2 | 1 | 1 | 2 | 2 |
| <https://youtu.be/odQOg-WZ7Eg?si=foVfwC4D9r74mDb6> | Haemorrhoid banding with Dallas colorectal surgeon | Haemorrhoids | 1 | 1 | 0 | 1 | 1 | 0 | 2 | 2 | 2 |
| <https://youtu.be/U-MyW-bbtRI?si=W9EUM3hSFgauUuZe> | Endoscopic band ligation of haemorrhoids | Haemorrhoids | 1 | 0 | 0 | 1 | 1 | 0 | 0 | 0 | 1 |
| <https://youtu.be/I6OmY-IX8_g?si=cx58nQO97GsmQUof> | Laser Haemorrhoidectomy | Haemorrhoids | 0 | 1 | 0 | 1 | 1 | 0 | 0 | 2 | 2 |
| <https://youtu.be/vRMX19hEBxQ?si=X0RN6R6v5laU_BjR> | LIS procedure for anal fissure/ surgeon Dr. Imtiaz Hussain | Anal fissure | 1 | 1 | 2 | 2 | 1 | 0 | 0 | 2 | 2 |
| <https://youtu.be/3Lly-RwT0No?si=SXiA3-j5uZOUwXGl> | Anal fissure lateral sphincterotomy at 5 OC | Anal fissure | 0 | 0 | 2 | 1 | 1 | 0 | 0 | 2 | 2 |
| <https://youtu.be/GeRzo_m1k-8?si=AmVRYII_a7qcVLGF> | Live laser fissurectomy in chronic fissure | Anal fissure | 1 | 1 | 1 | 2 | 2 | 2 | 0 | 2 | 2 |
| <https://youtu.be/fugUKhp5nlI?si=x-OaiA8MsG1ByZ1_> | Fastest live unedited surgery in 90 sec for intra anal condyloma wart by Dr Ashwin | Wart | 1 | 1 | 1 | 2 | 2 | 1 | 0 | 2 | 2 |
| <https://youtu.be/De-ynVJLD-A?si=OTUWSNaxgnTjslc3> | Management of anogenital warts part 2-fulgration of anal warts and peri-anal warts | Wart | 2 | 1 | 1 | 2 | 1 | 0 | 1 | 2 | 2 |
| <https://youtu.be/AjDaHQlSaFo?si=O3yiEntl3JBDw3f-> | Removal of rectal polyp laser surgery-Dr. Mir Asif | Polyp | 1 | 1 | 1 | 1 | 1 | 0 | 0 | 2 | 2 |
| <https://youtu.be/bcyf-O2jk2Q?si=MhbDklzuKf6jp6pR> | Laser sphicterolysis for fissures; laser haemorrhoidopexy for piles | Anal fissure | 2 | 2 | 1 | 2 | 2 | 0 | 1 | 2 | 2 |
| <https://youtu.be/HY9WcuRU5RY?si=qdnZ3LxmIb1rWl5_> | Anal fissure: closed lateral internal sphincterotomy | Anal fissure | 2 | 2 | 2 | 2 | 2 | 1 | 0 | 2 | 2 |
| <https://youtu.be/DgJUbhe-YGw?si=tLB53XyAE8YkN672> | Laser Surgery for peri anal fistula | Anal fistula | 2 | 0 | 1 | 2 | 2 | 0 | 0 | 2 | 2 |
| <https://youtu.be/r6CkRCDc7GI?si=mamXy3iwXg50vrtL> | Difficult to manage perianal fistulae and abscesses in Crohn's disease patients | Anal Fistula | 2 | 2 | 2 | 2 | 2 | 0 | 1 | 2 | 2 |
| <https://youtu.be/0Sj5TzvpWjg?si=9KW5T2n8sB7Jj4lm> | IBD Surgery: Perianal abscess and fistula | Fistula | 2 | 2 | 2 | 2 | 2 | 1 | 2 | 2 | 2 |
| <https://youtu.be/fu72-FGeBdo?si=kYdlESsal4SDfFh_> | Horseshoe fistula surgery using two different kind of setons | Fistula | 0 | 1 | 2 | 2 | 2 | 1 | 0 | 2 | 2 |
| <https://youtu.be/-Mn5VFOs0rM?si=5taVUVbCan8PxYHR> | Best Treatment of horseshoe shape fistula with supralevator abscess | Fistula | 1 | 2 | 2 | 2 | 2 | 2 | 0 | 2 | 2 |
| <https://youtu.be/p_LQGfj3EOA?si=ehubzSImAF5cfTuT> | Laser surgery of a blind anal fistula | Fistula | 1 | 1 | 2 | 2 | 2 | 0 | 2 | 2 | 2 |
| <https://youtu.be/WOdvWzMAJzI?si=xo1IcvSbodpV6ZIL> | Filac-Fistula Laser (Fistula removal) Tract Closure procedure by Biolitec | Fistula | 1 | 1 | 2 | 1 | 1 | 0 | 2 | 2 | 2 |
| <https://youtu.be/v83KDr_cXR0?si=UrEVIY6zu6r3osF_> | Anal Fistula: simple Fistulectomy | Fistula | 2 | 1 | 2 | 2 | 2 | 0 | 2 | 2 | 2 |
| <https://www.youtube.com/watch?v=g28akcpcWoQ> | Transanal Suture Mucopexy - a non-excisional cost-effective procedure for advanced hemorrhoids | Haemorrhoids | 1 | 2 | 2 | 2 | 1 | 0 | 0 | 2 | 2 |
| <https://www.youtube.com/watch?v=fQ18G5WIo-0> | Laser Hemorrhoidoplasty (LHP) - IBI Healthcare Institute | Haemorrhoids | 2 | 2 | 1 | 2 | 2 | 2 | 0 | 2 | 2 |
| <https://www.youtube.com/watch?v=o3YhMZIvx3s> | Hemorrhoid Surgery Unveiled: Outpatient vs. Inpatient – What You Need to Know | Haemorrhoids | 0 | 0 | 0 | 1 | 0 | 0 | 2 | 2 | 1 |
| <https://www.youtube.com/watch?v=qAI3T2EeLuw> | What is hemorrhoidectomy surgery? Get the REAL details here! \| Dr. Chung explains | Haemorrhoids | 0 | 0 | 1 | 1 | 1 | 0 | 1 | 0 | 1 |
| <https://www.youtube.com/watch?v=sfj6HSviVQI> | Stapled Hemorrhoidectomy for Prolapsing Hemorrhoids | Haemorrhoids | 0 | 0 | 0 | 1 | 0 | 0 | 2 | 2 | 1 |
| <https://www.youtube.com/watch?v=eC7rmWpG5Eg> | Stapled Haemorrhoidectomy using the PPH System - English | Haemorrhoids | 2 | 0 | 0 | 1 | 1 | 0 | 2 | 2 | 1 |
| <https://www.youtube.com/watch?v=l9BYk0zsrTQ> | The use of the gull retractor in Milligan Morgan hemorrhoidectomy | Haemorrhoids | 1 | 0 | 0 | 2 | 2 | 0 | 2 | 2 | 2 |

**Table S2: Non-peer reviewed videos along with scoring using modified assessment tools**
